# Red Blood Cell Alloimmunization in Pregnancy: A Review of the Pathophysiology, Prevalence, and Risk Factors

**DOI:** 10.7759/cureus.60158

**Published:** 2024-05-12

**Authors:** Sanusi Nurul 'Adani, Noor Suryani Mohd Ashari, Muhammad Farid Johan, Hisham Atan Edinur, Noor Haslina Mohd Noor, Mohd Nazri Hassan

**Affiliations:** 1 Hematology, School of Medical Sciences, Universiti Sains Malaysia, Kota Bharu, MYS; 2 Immunology, School of Medical Sciences, Universiti Sains Malaysia, Kota Bharu, MYS; 3 Forensic Programme, School of Health Sciences, Universiti Sains Malaysia, Kota Bharu, MYS

**Keywords:** hdfn, alloimmunization factors, laboratory methods, pregnant, alloimmunization, red blood cell

## Abstract

This review paper provides an overview of the risk factors and laboratory testing for red blood cell (RBC) alloimmunization in pregnancy. RBC alloimmunization is a significant medical issue that can cause haemolytic disease of the fetus and newborn (HDFN), leading to neonatal morbidity and mortality. Current HDFN prophylaxis targets only Rhesus D (RhD) alloimmunization, with no effective measures to prevent alloimmunization to other RBC antigen groups. Several factors can increase the risk of developing RBC alloimmunization during pregnancy, including fetomaternal haemorrhage, RBC and maternal genetic status, and previous transfusions. Identifying these risk factors is essential to execute the appropriate management strategies to minimize the risk of HDFN. The review also discusses the laboratory methods and overview of pregnancy management. The paper highlights the importance of identifying and managing the risk factors for RBC alloimmunization in pregnancy to minimize the risk of HDFN and improve neonatal outcomes.

## Introduction and background

Red blood cell (RBC) alloimmunization is the development of antibodies against non-self-antigens on RBC due to exposure, most commonly occurring after sensitization of blood transfusion or pregnancy [1,2]. In pregnancy, the production of maternal antibodies is significantly capable of reducing the life span of RBC antigen in the fetus, leading to serious alloimmune disorders, such as haemolytic disease of the fetus and newborn (HDFN) or erythroblastosis fetalis [3,4]. HDFN has been the main cause of neonatal and perinatal morbidity and mortality for many decades. The current prophylaxis to prevent HDFN is Rhesus immune globulin (RhIg), which came to medical use in the 1960s [5]. RBC alloimmunization rates among pregnant women have been drastically decreased and reported to be very low (ranging from 0.4% to 8.74%) after the implementation of RhIg prophylaxis [6]. Unfortunately, RhIg only offers protection against RhD alloimmunization and does not effectively prevent alloimmunization to other clinically significant RBC antigen groups, such as Kell, Duffy, and Kidd, which can also cause HDFN.

The exact pathogenesis of RBC alloimmunization in pregnancy is not well-understood beyond the presence of fetomaternal haemorrhage (FMH) and has not been widely explored. Previous studies found that human leucocyte antigen (HLA) or major histocompatibility complex (MHC) type, polymorphisms of immunoregulatory genes, immune activation status, and immune cell functional characteristics are the potential factors for RBC alloimmunization. However, the role of these factors in maternal alloimmunization remains not well-understood. This review provides an overview of the prevalence and factors influencing RBC alloimmunization in pregnancy. Identifying the risk factors of RBC alloimmunization is crucial for implementing appropriate management to minimize the risk of HDFN.

## Review

Pathophysiology of RBC alloimmunization

RBC alloimmunization is the immune response against foreign RBC antigens, commonly occurring following blood transfusions or during pregnancy [1]. Alloimmunization occurs as a response of the immune system to combat foreign RBC antigens. This process involves stimulation and differentiation of immune cells such as T cells and B cells. The immune system triggered the cells to proliferate and differentiate to encounter the RBC antigens. Antigen-presenting cells (APC) will digest and present antigens to activate T cells. Activated T cells will help B cells to differentiate and transform into plasma cells. These plasma cells will produce specific antibodies to mark RBC antigens for neutralization and destruction [7]. When pregnant women experience RBC alloimmunization, it can lead to significant clinical consequences for both the fetus and the mother, such as miscarriage, childbirth, and the most prominent effect, HDFN [8].

The risk of RBC alloimmunization during pregnancy occurs when the fetus inherits a different set of RBC antigens than the mother. For example, if the mother is RhD-negative and the fetus is RhD-positive, the mother's immune system may produce antibodies against the fetal Rh antigens, recognizing them as foreign [9]. The Rh antigen (D, C, c, E) is one of the RBC antigens that frequently engage in RBC alloimmunization during pregnancy [9].

Additionally, the type of immunoglobulin (Ig) produced by the immune system can impact the severity of the antigenic reaction. For instance, small antibodies such as IgG are clinically significant as they have the capability to cross the placenta compared to IgM [10]. As Rh antibodies belong to IgG class, typically IgG1 or IgG3, these kinds of antibodies are able to cross the placenta and target fatal RBCs, causing RBC destruction and HDFN [11]. Incompatibility due to the ABO group is also common, but the severity is not significant due to the large size of IgM antibodies, restricting the capability of antibodies to cross the placenta [12].

Apart from alloimmunization, the underlying disease in patients may contribute to the impairment of immune cells, affecting the health condition of the patients. Compared to non-alloimmunization sickle cell disease (SCD) patients, those with alloimmunization show an increase in the number of T cells and B cells [13-15]. In polytransfused patients, B cell numbers show an increase, while T cell numbers decrease [2].

Although the pathogenesis of RBC alloimmunization has been extensively studied in regular transfused patients and animal models, it has not yet been thoroughly investigated in pregnant women. Possible factors that may contribute to RBC alloimmunization include genetic factors such as MHC or HLA type and polymorphisms of immunoregulatory genes, immune activation status, functional characteristics of immune cells, prior exposure to antigens, and various other factors [16-18].

Prevalence of RBC alloimmunization in pregnancy

The reported rate of alloimmunization in the general population ranges from 0.46% to 2.4% [9]. Besides pregnancy, patients who received transfusions are among the most affected by alloimmunization. Individuals with conditions such as SCD, and thalassemia, and those who undergo haemotheraphy are exposed to multiple antigens from donor sources, leading to the development of alloimmunization. In these populations, female patients commonly exhibited a higher percentage of alloimmunization compared to males due to pregnancy and delivery exposure as an important independent risk factor (Table 1).

**Table 1:** Frequency of RBC alloimmunization between genders among transfused patients.

Patient group	Total patient (N)	Total alloimmunized patient (n)	Male, n (%)	Female, n (%)	Author (year)
SCD	50	31	14 (45.2)	17 (54.8)	Siransy et al. (2018) [19]
Transfusion-dependent β-thalassemia	268	25	11 (44.0)	14 (56.0)	Al-Riyami et al. (2018) [7]
Multi-Transfused, Oncology	8115	18	1 (5.5)	17 (94.5)	Mangwana et al. (2019) [13]
Chronic kidney disease	249	31	12 (38.7)	19 (61.3)	Yusoff et al. (2020) [15]
Haemotheraphy	11253	179	35 (19.5)	144 (80.5)	Barbosa et al (2022) [14]
SCD	556	107	46 (43.0)	61 (57.0)	AlDawood (2023) [20]
Total	20491	391	109 (28.6)	272 (71.4)	

The underlying disease, frequency of transfusions, and sample size also play crucial roles in contributing to the prevalence of alloimmunization in the population. RBC alloimmunization rates among pregnant women have been extensively studied in many areas around the world, with the frequency being found to range from 0.4% to 8.74% worldwide (Table 2).

**Table 2:** Prevalence of RBC alloimmunization in pregnant women in different countries.

Country	Total number of screened pregnant women (N)	Number of pregnant women with antibodies (n)	Overall prevalence (%)	Most prevalence antibodies (%)	Author (year)
Sweden	11350	629	0.57	Anti-Lea (20.3), anti-D (19.4), anti-Bg (11.8)	Filbey et al. (1995) [21]
United Kingdom	22264	244	1.0	Anti-D (41), anti-K (17.2), anti-E (12)	Howard et al. (1998) [22]
Ireland	34913	186	0.53	Anti-C (26), anti-K (22), anti-c (12)	Chandrasekar et al. (2001) [23]
China	28303	230	0.79	Anti-M (57.6), anti-E (19.7), anti-S (10.6)	Lee et al. (2003) [24]
Sweden	78145	316	0.4	Anti-D (60), anti-Fya (10), anti-C (7)	Gottvall et al. (2008) [6]
India	3577	45	1.25	Anti-D (78.4), anti-C (11.76), anti-M (3.92)	Pahuja et al. (2011) [25]
Turkey	4840	65	8.74	Anti-D (69.2), anti-Lea (10.76), anti-K (3.07)	Altuntas et al. (2013) [26]
India	5347	79	1.48	Anti-D (34.2), anti-Lea (10.1), anti- Leb (7.6)	Varghese et al. (2013) [27]
Czech	45435	683	1.5	Anti-E (5.7), anti-D (4.0), anti-M (1.5)	Holusková et al. (2013) [28]
Pakistan	1000	18	1.8	Anti-M (15); anti- Lea (15), anti-C (5)	Karim et al. (2015) [29]
Malaysia	5163	51	0.99	anti-E (13.73), anti-Lea (9.8), anti-D (5.88)	Hassan et al. (2015) [30]
Australia	66354	482	0.73	Anti-E (27.6), anti-D (10.4), anti-K (9.5)	Pal et al. (2015) [31]
India	1000	7	0.7	anti-E (85.7), anti-c (71.4), anti-Cw (14.3)	Sankaralingam et al. (2016) [32]
Spain	27609	176	0.63	Anti-D (24), anti-E (20), anti-C (13)	Solves et al. (2017) [33]
Iceland	87437	917	1.04	anti-M (19.4), anti-E (19.0), anti-D (12.5)	Bollason et al. (2017) [34]
India	1960	20	1.02	Anti-D (90), anti-c (5), anti-H (5)	Kahar (2018) [35]
United States	4545	440	0.74	anti-E (38.2), anti-K (20.6), anti-M (17.6)	Moinuddin et al. (2019) [36]
India	530	12	2.3	Anti-D (56.25), anti-C (37.5), anti-E (6.25)	Naik et al. (2020) [37]
India	8920	432	4.84	Anti-D (92.85), anti-C (10), anti-E (3.57)	Dholakiya et al. (2021) [38]
India	2084	65	3.1	Anti-D (83), anti-M (9.23), anti-C (3.08)	Gothwal et al. (2022) [39]
Serbia	25694	761	2.96	anti-D (23.34), anti-M (11.85), anti-E (9.41)	Bujandric et al. (2022) [40]

A common complication of RBC alloimmunization in pregnant women is FMH, which may have devastating consequences to the fetus such as fetal demise, neurologic injury, hydrops, delivery of a severely anaemic infant and stillbirth [41-43]. There are more than 60 different RBC antigens, which are commonly referred to as alloimmunization, especially the Rhesus, Kell, Duffy, Kidd, and MNS blood group systems [38].

However, the frequencies of RBC antibodies are reported to vary among populations and ethnic groups. The differences in alloimmunization among populations and ethnic groups may be due to genetic variations in the immune system and the distribution of antigens. Certain ethnic groups may have a higher frequency of certain antigens, while others may have a lower frequency. In Asian countries, antibodies toward Rhesus antigen showed the highest prevalence with anti-E and anti-D, respectively [4,30,37,38], while, among Caucasian, anti-K is the most common [22,23,26,30,31,36,44]. This means that individuals from different ethnic groups may be more or less likely to develop alloimmunization in response to exposure to certain antigens.

**Figure 1:**
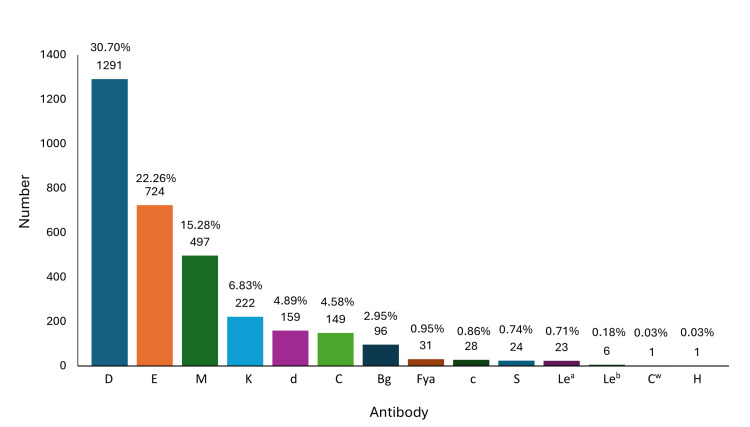
Prevalence of antibodies among alloimmunized pregnant women from 1995 to 2022. Anti-D showed the highest antibody that causes alloimmunization among alloimmune patients compared to another antibody due to inappropriate application of RhIg prophylaxis.

A prevalence study from 1995 until 2022 showed that anti-D is the most common alloantibody that causes alloimmunization in pregnant women even when RhIg prophylaxis is available as prevention indicates that rhesus antigen is the most clinically significant RBC antigen that frequency engages with RBC alloimmunization during pregnancy (Figure 1).

The current, most recommended policy in many countries prescribes RhIg to non-sensitized RhD-negative pregnant women in the 28th week of gestation onwards [13,45,46]. Administering RhIg too late may not effectively prevent the formation of anti-D antibodies. As gestation progresses, the amount of fetal blood crossing the placenta also increases, which can make the administered amount of RhIg insufficient to provide protection against anti-D antibody formation. Failure to receive the full recommended dose of RhIg or not following up with medical care after exposure to Rh-positive blood may increase the risk of developing anti-D antibodies.

Screening for RBC alloimmunization in pregnancy

Laboratory methods for the detection of RBC alloimmunization are often part of a comprehensive approach. The detection of RBC alloimmunization is important for determining the risk of HDFN, which is a rare but potentially severe event. The specific methods applied may vary based on the individual case and available resources in the healthcare setting.

ABO/RhD Typing and Antibody Screening

ABO and RhD typing of the mother are routinely performed at the first prenatal visit to identify potential incompatibilities that may lead to alloimmunization. The presence or absence of ABO and RhD antigens on RBCs will rely on an agglutination reaction due to the interaction of known antibodies and parent’s antigens. This test may be repeated in the late second trimester if needed [47]. Besides ABO and RhD typing, antibody screening by indirect antiglobulin test (IAT) is also a routine screening that will perform after blood typing to identify maternal antibodies against RBC antigens that can cause a risk to the fetus. This test also may be repeated more frequently if have a risk of alloimmunization [48]. Antibody screening also may be repeated at 28 weeks to detect new alloimmunization [47].

Antibody Identification and Titration

Following the presence of antibodies for antibody screening tests during pregnancy, the antibody identification test plays a crucial role in further elucidating the specific antibody involved and its potential impact on the fetus. By performing the antibody identification test, specific antigens targeted by the antibodies will be identified; thus, the clinical significance (anti-D, anti-K, anti-E) of the involved antibody and risk of HDFN can be assisted [36,48]. After a specific antibody is identified, antibody titration should be tested. This testing is crucial to assess the levels of antibodies present and their potential impact on the fetus, particularly in cases of HDFN due to maternal-fetal blood group incompatibility [48]. While a high antibody titre is often associated with severe HFDN, it's not the major factor. The specific type of antibody involved also impacts the severity of the condition. As reported from a previous study, anti-K is the type of antibody that has a lower titre but can contribute to the critical impact compared to anti-D [49]. The antibody titration test is typically repeated every two to four weeks when the pregnancy is identified with clinically significant antibodies [47]. For clinically non-significant antibodies (anti-M, anti-Jka, anti-Le), a general precaution might involve a repeat screening around second to third trimester of pregnancy [47,50].

Fetal Blood Sampling (FBS)

**Figure 2:**
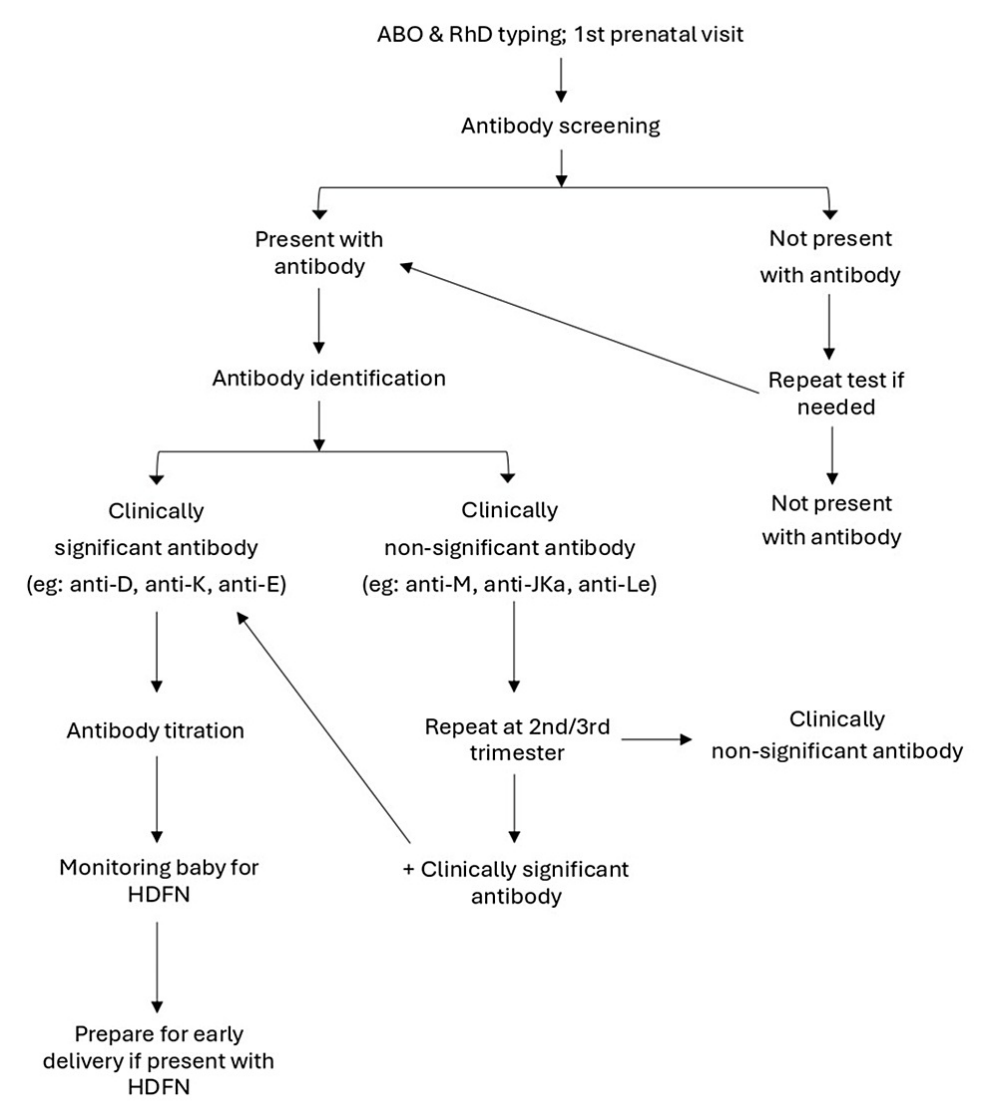
The flowchart provides a simplified overview of screening and detection of alloimmunization in pregnancy starting from the first prenatal visit until delivery. ABO and RhD typing will be screened, followed by antibody screening. If the mother presents with an antibody, the type and significance of the antibody will be identified and will be continuously monitored until delivery.

In the case of the alloimmunized mother, if the case needs a comprehensive analysis of fetal health and blood parameters, including Rh status, anaemia severity, and potential infections, FBS will be performed. This test is performed via cordocentesis, where a needle is inserted through the mother's abdomen and into the umbilical cord under ultrasound guidance. This test will be conducted for high-risk situations with evidence of potential fetal compromise, where information from other tests such as ultrasound monitoring is insufficient [51]. Figure 2 shows the flowchart of screening and detection of alloimmunization in pregnancy.

Factors influencing RBC alloimmunization in pregnancy

Prior Antigen Exposure

The primary factor influencing alloimmunization is blood transfusion and pregnancy [52]. If a woman receives blood with incompatible antigens, her immune system will develop memory cells and antibodies against those antigens. Comparably, during miscarriage or delivery, even without complications, small amounts of fetal blood can enter the maternal circulation and trigger the immune system if the fetus possesses an incompatible antigen.

This sensitization can lead to either single alloimmunization or multiple alloimmunization, depending on the exposure history, and significantly impacts future pregnancies. Once the immune system remembers antigens from previous exposure, the subsequent exposure will trigger a faster and stronger response.

Multiple alloimmunizations are more significant compared to single alloimmunization due to the increased complexity and potential clinical implications associated with developing antibodies against multiple RBC antigens [2]. When an individual experiences multiple alloimmunizations, difficulty identifying compatible blood units, and higher transfusion are required for the fetus or newborn. For example, a woman with anti-RhD and anti-Kell antibodies would require blood negative for both antigens, thus significantly narrowing compatible donor options.

Fetomaternal Hemorrhage

During pregnancy, a common obstetrical event called FMH occurs where a small amount of fetal blood cells enters to the maternal bloodstream. Usually, this event happens without any maternal or fetal consequences [53]. However, in some cases, larger amounts of fetal blood can cross the placental barrier and enter the maternal circulation due to trauma (maternal falls or motor vehicle accidents), invasive procedures during pregnancy (amniocentesis or chorionic villus sampling, abortion), miscarriage, and intrauterine infection [54-56]. Furthermore, around the time of delivery, pregnant women are exposed to fetal RBCs, which is also one of the factors that contribute to alloimmunization [52].

In developing countries, RhIg prophylaxis is given to Rh-negative mothers at 30 weeks of gestation and 48 hours of delivery or after an invasive prenatal procedure to prevent an alloimmunization reaction towards Rh-positive fetus. Despite adequate antenatal and postnatal RhIg prophylaxis, postnatal events such as caesarean section, manual removal of the placenta, excessive postpartum haemorrhage, delivering at or after 42 weeks of gestation, and perinatal death are still found as the risk factors to alloimmunization occur [56,57]. FMH volume testing and extra prophylaxis shots may be required after the postnatal procedure.

As a modern approach, molecular determination of fetal RhD through maternal plasma known as non-invasive prenatal testing (NITP) can provide pregnancy safety as NIPT can be done earlier in pregnancy, allowing the detection of alloimmunization without the risk of FMH [58-60]. This testing can determine the fetal RhD status by real-time polymerase chain reaction using cell-free DNA obtained from maternal plasma [58,60]. As the detection of fetal RhD phenotype is sensitive during the third trimester, the result of this test can be a guide to deliver the targeted anti-D prophylaxis during antenatal and postnatal, thus reducing the unnecessary treatment and leading to cost savings [60].

Mothers with a history of blood transfusions in the past may have produced antibodies against foreign blood group antigens. If the fetus inherits these antigens from the father, the mother may produce antibodies against them [12]. These antibodies can enter the placenta and harm the red blood cells of the developing fetus, leading to HDFN, which can cause a variety of complications, such as anaemia, jaundice, and, in severe instances, brain injury and even death [61].

RBC Antigen

Besides procedures or events during prenatal or postnatal, the RBC antigen itself is also one of the risk factors that contribute to RBC alloimmunization in pregnant women. The ability to present the RBC antigen to the immune system is known as immunogenicity, which also depends on the type of antigen and the individual's genetic makeup [52].

The antigen copy number is also related to immunogenicity as a low number of antigen copy number reduces the ability to mount antigen to the immune system; thus, the immune system is unable to generate alloantibodies. In the context of pregnant alloimmunization, the immunogenicity and antigen copy number of the fetal RBC antigen can all play a role in determining whether the mother will produce antibodies against the antigen. Highly immunogenic antigens with high copy numbers are more likely to stimulate an immune response and lead to the production of antibodies. An example is M antigen with a copy number of 353,000, making it one of the antigens with high immunogenicity and causing haemolysis and subsequent complications, such as anaemia in the fetus [62,63].

While the density of antigens on RBCs refers to the number of antigen molecules on the surface of each RBC, antigen density can vary depending on the antigen zygosity [52,64]. For example, individuals who are homozygous for the D antigen (R1R1, R2R2) express nearly twice the number of D antigens compared to those who are hemizygous (R1r, R2r) for the D antigen.

When exposed to incompatible blood group antigens, the body produces numerous alloantibodies, which can cause a significant clinical implication. The specific types of alloantibodies are determined by the antigens to which the individual has been exposed. Some of the common types of alloantibodies identified in pregnant women include anti-D, anti-C, anti-K, anti-M, and anti-E [36]. These alloantibodies can lead to HDFN and other complications, emphasizing the importance of identifying and monitoring the presence of these antibodies during pregnancy. The prevalence and specificity of clinically significant red cell alloantibodies in pregnant women have been studied, with anti-D, anti-K, anti-M, anti-C, and anti-E being among the most frequently identified alloantibodies [36].

Alongside assessment of antigen expression on RBC using the phenotyping method, the prediction of the antigen expression using the genotyping method allows for more accurate prediction of alloimmunization risk, thus minimising the risk of complications and giving treatment to the needs [52]. Table 3 summarises the molecular method used to predict RBC antigen expression by using the genotyping method.

**Table 3:** Summary of molecular methods used to identify RBC antigens of alloimmune and their advantages and disadvantages.

Method	Purpose	Advantages	Limitation	Citations
Polymerase chain reaction (PCR) real time and QF (quantitative fluorescence)	To amplifies specific DNA regions linked to RBC antigens	Highly sensitive and specific	More expensive than serological methods	Bohmova et al. 2020 [65]
Single-nucleotide polymorphism (SNP) genotyping	To identifying variations in genes that control RBC antigens	Highly specific and detects wider range of antigens	Expensive and requires specialized equipment	Shi et al. 2019 [66]
Sanger sequencing	Determine the exact sequence of nucleotides in a specific region RBC antigen	Provides highly accurate sequencing data for the targeted region	Only analyse one target region at a time	Chang et al. 2020 [67]
Next-generation sequencing (NGS)	To identify the targeted RBC antigen and uncover any unexpected variations in immune response genes	Most comprehensive method, detecting all known and potentially unknown antigens	Not widely available	Khandelwal et al. 2023 [68]

Immunomodulatory and Genetic

During pregnancy, a pregnant woman's immune system may recognize fetal red RBC antigens as foreign if they differ from her own. This can trigger an immune response called alloimmunization, where the mother's body produces antibodies against the fetal RBC antigens. Factors such as immunomodulatory processes and a woman's genetics can significantly influence the likelihood and severity of alloimmunization.

Immunomodulatory and genetics may influence alloimmunization in pregnant women. Human leukocyte antigen (HLA) plays an important role in presenting antigens to T-cells. Due to HLA restriction, some antigens cannot be presented to T-cells, which can impact the production of antibodies. The term ‘non-responder’ can be used for the mother with HLA restriction since the mother fails to generate detectable alloantibodies to RBC antigens after exposure [69]. HLA restriction study may provide insight by preventing certain HLA, preventing the alloantibodies expression, and then promoting safe transfusion and pregnancy [52,70]. Specific HLA types, such as HLA-DQB1*0602, are associated with an increased risk of Rh D alloimmunization [71].

In pregnant women, dendritic cells (DCs) will encounter fetal RBCs that have crossed the placenta during pregnancy. DCs act as APC captures and processes foreign RBC antigens and present them to T cells in conjunction with specific HLA molecules. The specific HLA-antigen complex determines how the T cell recognizes the antigen [2]. Depending on the presented antigen and HLA context, different T cell subsets become activated. For example, Th1 cells will promote cellular immunity, while Th2 cells, activated by specific cytokines such as IL-4 and IL-6, will become central to antibody production. They secrete cytokines (IL-5, IL-10) that stimulate B cells to proliferate and differentiate into plasma cells [2,72].

B lymphocytes also act as APC recognizing the foreign fetal RBC antigen through their B cell receptors (BCRs), and some B lymphocytes will proliferate and become plasma cells [73,74]. These antibody factories produce large amounts of Ig, primarily IgG antibodies, specifically targeting the fetal foreign RBC antigens. These antibodies can cross the placenta and attack fetal RBCs, potentially leading to complications such as haemolytic anaemia in severe cases [10].

**Figure 3:**
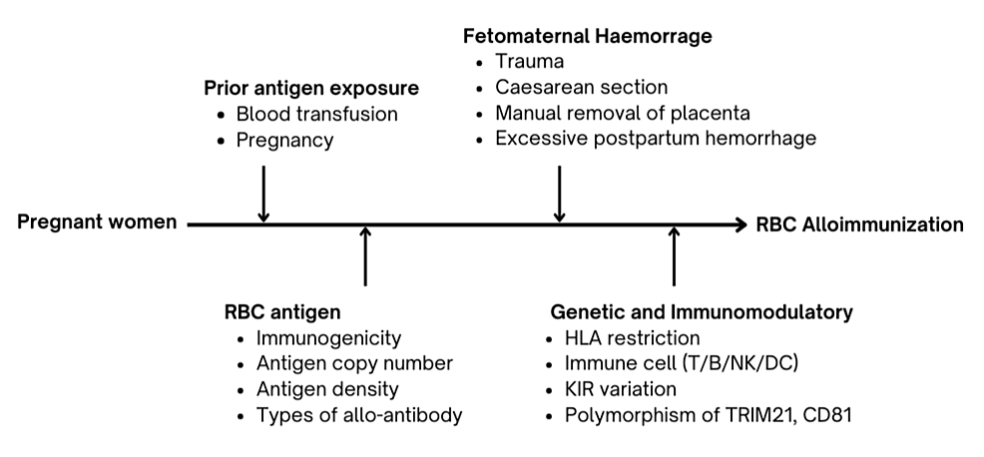
Factors that influence RBC alloimmunization in pregnant women. The figure illustrates the factors that influence RBC alloimmunization in pregnant women, a condition where an individual develops antibodies against foreign RBC antigens. This can occur following exposure to antigens through blood transfusions or pregnancy and other complex processes influenced by multiple factors on fetomaternal haemorrhage, RBC antigen, genetic and immunomodulatory.

Natural killer (NK) cells can eliminate cells that present fetal cells that present with foreign HLA molecules, thus influencing the overall immune response [75]. The variations of killer cell immunoglobulin-like receptors (KIRs) have been linked to susceptibility or resistance to alloimmunization [76]. While cytokines become the chemical messengers that regulate the immune response, specific cytokines may influence T cell activation, B cell proliferation, and antibody production. For example, IL-4 secreted by Th2 is associated with type 2 immune responses and promotes B cell proliferation and IgE production [77]. IL-10, on the other hand, is an immunoregulatory cytokine produced by B cells that can have both anti-inflammatory and regulatory effects on T cells [78]. Figure 3 summarizes the factors that influence RBC alloimmunization in pregnant women.

## Conclusions

RBC alloimmunization during pregnancy is common, but this complication can have a negative impact on both mother and fetus. Depending on the population investigated and the screening techniques used, the frequency of RBC alloimmunization in pregnancy can vary considerably. An ongoing assessment of the mother's antibody levels, and fetus condition is necessary for RBC alloimmunization management during pregnancy. Approaches such as intravenous immunoglobulin or intrauterine transfusions are preventative measures to reduce the likelihood of unfavorable consequences.
